# A Probabilistic Digital Twin for Leak Localization in Water Distribution Networks Using Generative Deep Learning

**DOI:** 10.3390/s23136179

**Published:** 2023-07-05

**Authors:** Nikolaj T. Mücke, Prerna Pandey, Shashi Jain, Sander M. Bohté, Cornelis W. Oosterlee

**Affiliations:** 1 Centrum Wiskunde & Informatica, Science Park 123, 1098 XG Amsterdam, The Netherlands; sbohte@cwi.nl; 2 Mathematical Institute, Utrecht University, 3584 CS Utrecht, The Netherlands; c.w.oosterlee@uu.nl; 3 Department of Management Studies, Indian Institute of Science, Bangalore 560012, India; preranap@iisc.ac.in (P.P.); shashijain@iisc.ac.in (S.J.); 4 Swammerdam Institute of Life Sciences (SILS), University of Amsterdam, 1098 XH Amsterdam, The Netherlands; 5 Bernoulli Institute, Rijksuniversiteit Groningen, 9747 AG Groningen, The Netherlands

**Keywords:** leak localization, water distribution network, Bayesian inverse problems, generative deep learning, digital twin

## Abstract

Localizing leakages in large water distribution systems is an important and ever-present problem. Due to the complexity originating from water pipeline networks, too few sensors, and noisy measurements, this is a highly challenging problem to solve. In this work, we present a methodology based on generative deep learning and Bayesian inference for leak localization with uncertainty quantification. A generative model, utilizing deep neural networks, serves as a probabilistic surrogate model that replaces the full equations, while at the same time also incorporating the uncertainty inherent in such models. By embedding this surrogate model into a Bayesian inference scheme, leaks are located by combining sensor observations with a model output approximating the true posterior distribution for possible leak locations. We show that our methodology enables producing fast, accurate, and trustworthy results. It showed a convincing performance on three problems with increasing complexity. For a simple test case, the Hanoi network, the average topological distance (ATD) between the predicted and true leak location ranged from 0.3 to 3 with a varying number of sensors and level of measurement noise. For two more complex test cases, the ATD ranged from 0.75 to 4 and from 1.5 to 10, respectively. Furthermore, accuracies upwards of 83%, 72%, and 42% were achieved for the three test cases, respectively. The computation times ranged from 0.1 to 13 s, depending on the size of the neural network employed. This work serves as an example of a digital twin for a sophisticated application of advanced mathematical and deep learning techniques in the area of leak detection.

## 1. Introduction

Water distribution systems make up a large and important part of our civil infrastructure. They need to be safe, efficient, and reliable. Ensuring a constant supply of clean water is, however, not an easy task. Distribution is typically carried out using networks of pipes, which can be highly intricate and involve multiple components, such as several kilometers of pipe segments and numerous junctions, valves, pumps, reservoirs, and tanks. Such networks are difficult to manage and they are prone to failure due to leakages and blockages, which may result in economic losses and environmental damage. Therefore, it is important to have a quick and trust-worthy monitoring method in place. Monitoring is typically done by recording information from a number of sensors installed at critical locations within the network. However, detecting the occurrence and location of leaks can still be a challenging task, even with sensor data being available, because the information captured by sensors will be incomplete in both time and space, making it difficult to pinpoint the exact location and timing of the leak. In this paper, we address the problem of leak localization in real time by means of machine learning techniques.

For a leak localization framework to be considered useful in practice, it has to satisfy certain requirements. First, it should be sufficiently accurate. Second, it must be computationally fast, so that any leakage can be identified quickly. Third, it must be reliable. That is, the framework should not only provide an estimated leak location but also a measure of the level of confidence of the estimate. Fourth, it should be general and work under different circumstances. The framework should be sufficiently generic to work with different water distribution networks with widely varying complexities. Last, it must be flexible. In this context, flexibility refers to various aspects, such as whether it is possible to include prior knowledge when such becomes available, and whether it can handle different kinds of sensor readings or varying numbers of sensors, etc. There are not many approaches that satisfy all these requirements, since many of them are difficult to satisfy simultaneously. For example, computing uncertainty often comes at the cost of computation time, and generalized models are typically less accurate than models tailored to specific cases, and they struggle with incorporating prior knowledge, as they then lose their general nature. The framework presented here satisfies all these requirements, at the cost of a computationally intensive training stage.

### 1.1. Leak Localization Literature

There has already been a significant amount of research in the area of leak localization. However, not many approaches satisfy all the above-mentioned requirements. The authors in [1,2] made use of a model to generate synthetic sensor observations. The residuals between the model output and the observed values were then used to predict the leak location using a trained classifier. Similarly, in [3] residuals were employed for leak detection, after which the characteristics of the residuals were used to localize the leak. In [4], encoding of sensor observations was used, together with a trained classifier and a graph theory-based clustering method. While these approaches are computationally fast and were shown to be accurate for the selected test cases, they mainly work for the sensor configurations they were trained on and are limited regarding uncertainty quantification, as they do not model the inherent input uncertainty in, e.g., the demand. Furthermore, even if it is available, prior information cannot be embedded in these approaches. On the other hand, in [5], a combination of model- and graph theory-driven techniques was used, together with online training of a neural network classifier, to predict the cluster of nodes in which the leak was present. This is a flexible setup that allows for changes in sensor configurations. However, the uncertainty quantification is still limited, as only the size of the predicted cluster is used as a proxy for the reliability of the prediction.

### 1.2. Literature on Modern Machine Learning Techniques

In a rather different setting, the authors of [6] proposed a GAN-based automatic property generation approach, to generate verification properties for model checking. The verification properties, encoded in computational tree logic, were used as input to the GAN, whereas [7] presented a novel memory-augmented autoencoder approach for unsupervised anomaly detection in IoT data, which mitigated over-generalization by incorporating a memory mechanism in a time-series autoencoder (TSMAE). A methodology based on semi-supervised learning was introduced in [8], using an opposition-based novel updating spotted hyena optimization (ONU-SHO)-based recurrent neural network (RNN) for handling continuous or streaming data. The authors in [9] proposed a new record linkage (with the task of identifying and linking records from multiple sources) model for unstructured data, wherein a deep learning approach is used to improve the generalization of the Siamese multilayer preceptron model, to make it less sensitive to parameter selection.

### 1.3. Bayesian Inference

As an alternative to the above-mentioned approaches, one can make use of Bayesian inference. This allows one to accurately solve the leak localization problem using uncertainty quantification. The general technique is to compute the data likelihood and combine this with a prior. The output from a Bayesian inference approach is then a distribution of the leak location conditioned on sensor observations, i.e., the posterior distribution. This approach also provides flexibility, as new sensor configurations can be incorporated by simply modifying the likelihood. Both the sensor noise and model uncertainty are modeled within the likelihood. That is, the uncertainty associated with imperfect sensors and, e.g., stochastic nodal demand in the water network, can be included in the computation of the posterior directly. Furthermore, one can incorporate prior information in a straight-forward manner. However, the Bayesian approach has the significant drawback of being computationally expensive. Approximating the likelihood accurately requires solving the model many times, due to the nonlinearity and high-dimensionlity. Hence, it is often infeasible to run a Bayesian inference procedure in real time. In [10], the authors overcame this problem by assuming normally distributed demands and inputs, to be able to use gradient-based optimization together with kernel methods. However, this assumption may be restrictive. Instead, we propose an alternative solution to this problem that does not require such a restriction.

### 1.4. Computational Bottleneck

To overcome the computational bottleneck associated with Bayesian inference, one can make use of a surrogate model. A surrogate model is trained in an offline stage, before being used in an online stage for leak localization. The usage of surrogate modeling has become widespread nowadays, due to the potential for computational speed-up without an essential loss of accuracy. Conventionally, a linear dimensionality reduction, such as proper orthogonal decomposition (POD), is used together with a regression method in the reduced space; for example, in [11], a combination of POD and radial basis functions with neural networks was used in an inverse analysis for structural diagnosis. In [12], POD was used with stochastic spectral methods to relate the input to the output within a Bayesian inverse problem to speed up the process. In [13], POD was combined with Gaussian processes, to speed up nonlinear structural analysis. In recent years, deep neural networks have become a popular choice for surrogate models in scientific computing, due to their performance in dimensionality reduction and their predictive power [14,15,16]. For the purpose of speeding up Bayesian inference, we decided to model a high-dimensional stochastic problem. We made use of the concept of generative modeling. While applications of generative modeling in various scientific fields are already widespread [17,18,19], it has not yet been tailored to the area of water management. The general concept is to train a neural network to learn the underlying distribution of a data set, in order to be able to sample from it after a training phase. This enables fast sampling from complicated and highly dimensional distributions. There are several kinds of generative neural network, such as generative adversarial networks [20], variational autoencoders [21], and diffusion models [22]. Each of these models has its advantages and drawbacks. Specifically, there are three factors to consider when using generative models: sampling quality, speed, and diversity [23]. In this work, we adopt the Wasserstein autoencoder [24], as it performs highly satisfactory in these three criteria.

### 1.5. Overview of the Paper

In this paper, we present a novel leak localization framework based on Bayesian inference and generative deep learning. By formulating the leak localization problem as a Bayesian inverse problem, the uncertainty in model parameters and sensor observations is included in the leak location estimation, which enables accurate uncertainty quantification of the predicted leak location. Furthermore, prior information can be included in the computations. To overcome the computational drawbacks, we make use of neural networks as the stochastic surrogate model. A neural network is trained as a generative model to estimate the distribution of pressure heads and flow rates given a certain leak location.This enables the fast evaluation of the likelihood function, while retaining a high accuracy.

In Section 2, we present the theory behind our framework. The problem setting and the underlying equations are described; the leak localization problem is presented as a Bayesian inverse problem; and the generative neural networks, WAEs, and neural network architectures are presented. In Section 3, we combine the components from Section 2 in the presented framework. In Section 4, we showcase the framework on three test cases. The paper is then concluded in Section 5.

## 2. Problem Setting and Preliminaries

In this section, we introduce the problem setting and briefly cover the preliminaries for the proposed framework. We start by introducing the mathematical model for the water distribution network, which allows us to simulate the pressure heads and flow rates given a set of parameters, such as the demand, pipe roughness, and leak location. Then, we describe leak localization as a Bayesian inverse problem and explain how the Bayesian setting allows us to model the uncertainty and incorporate prior information. Furthermore, we state the assumptions and the drawbacks of the proposed framework. Lastly, we present the relevant machine learning framework and deep learning architecture. Specifically, we discuss Wasserstein autoencoders, which are crucial for speeding up the Bayesian inference, as well as residual and transformer neural networks, which enable us to replace the conventional mathematical model, without losing significant accuracy.

### 2.1. Problem Setting

We consider a water distribution network that has $N_{p}$ pipes, $N_{j}$ variable head nodes, and $N_{f}$ fixed head nodes. The head losses in all pipes in the network are assumed to be modeled by the Hazen–Williams formula, so the relation between the heads at two ends (nodes *i* and *k*) of a pipe *j* and the flow is given by

(1) $$h_{i}-h_{k}=r_{j}Q_{j}^{n},$$

where $Q_{j}$ is the flow rate in pipe $p_{j}$; $h_{i}$ is the head at node *i*, n=1.852, and $r_{j}$ is the pipe resistance factor, which depends on the length, diameter, and the material of the pipe. Define $q={\left(Q_{1},\ldots ,Q_{N_{p}}\right)}^{⊤}$ as a vector of unknown fluid flow rates in the pipes.

The network topology is modeled using the incidence matrices $A_{1}\in R^{N_{p}\times N_{j}}$ and $A_{2}\in R^{N_{p}\times N_{f}}$ for the unknown head nodes and the fixed head nodes, respectively. These incidence matrices are defined as

(2) $$A_{b}=\left\{\begin{array}{cc} -1 & \mathrm{if}\;\mathrm{the}\;\mathrm{flow}\;\mathrm{in}\;\mathrm{pipe}\;j\;\mathrm{enters}\;\mathrm{the}\;\mathrm{node}\;i,\\ 0 & \mathrm{if}\;\mathrm{the}\;j\;\mathrm{does}\;\mathrm{not}\;\mathrm{connect}\;\mathrm{to}\;\mathrm{the}\;\mathrm{node}\;i,\\ 1 & \mathrm{if}\;\mathrm{the}\;\mathrm{flow}\;\mathrm{in}\;\mathrm{pipe}\;j\;\mathrm{leaves}\;\mathrm{the}\;\mathrm{node}\;i, \end{array}\right.$$

where b=1,2. The unknown heads at different nodes are defined as $h={\left(h_{1},\ldots ,h_{N_{j}}\right)}^{⊤},$ the known nodal demands as $d_{m}\in R^{N_{j}}$, and $e_{l}\in R^{N_{f}}$ are the fixed head elevations. Additionally, we define the following matrices: *O*, as the $N_{j}\times N_{j}$ zero matrix, o, as an $N_{p}\cdot N_{j}$ zero vector, and an $R^{N_{p}\times N_{p}}$ diagonal matrix G(q), with diagonal entries,

(3) $$\begin{array}{c} G_{jj}\left(q\right)=r_{j}{\left|Q_{j}\right|}^{n-1}. \end{array}$$

The hydraulic problem entails solving the unknown flow rates in the $N_{p}$ pipes, q, and the unknown heads at the $N_{j}$ nodes, h, given the network topology $A_{b}$, the demand at the nodes $d_{m}$, and a fixed head elevation, $e_{l}$, such that the mass and energy for the flow are balanced. The continuity equation to be solved in matrix form is written as (see [25] for details):

(4) $$f\left(x\right)=\left(\begin{array}{cc} G(q) & -A_{1}\\ -A_{1}^{⊤} & O \end{array}\right)\left(\begin{array}{c} q\\ h \end{array}\right)-\left(\begin{array}{c} A_{2}e_{l}\\ d_{m} \end{array}\right)=o,$$

where we solve for the unknown vector $x:={\left(q^{T},h^{T}\right)}^{T}.$ The above set of equations is typically solved using Rossman’s popular program EPANET ([26]), to obtain a steady state solution.

A leak is modeled by adding a leak demand at a specific node. The leak demand is given by

(5) $$\begin{array}{c} d_{\mathrm{leak}}=C_{d}Ap^{\alpha }\sqrt{\frac{2}{\rho }}. \end{array}$$

Typically, α=0.5 is chosen [26]; $C_{d}$ is the dimension-less discharge coefficient, $A\;[m^{2}]$ is the leak area, $g\;[{m/s}^{2}]$ is acceleration by gravity, p[Pa] is the gauge pressure, and $\rho \;[{\mathrm{kg}/m}^{3}]$ is the density. Note that a leak is modeled as a nodal demand dependent on the pressure head. However, in the vast majority of cases, a leak will be located in a pipe section and not at a node. Therefore, we will introduce an extra node into the pipe section in which the leak occurs. The demand on that node is the leak demand.

### 2.2. Leak Localization as a Bayesian Inverse Problem

Detecting water leaks can be formulated as an inverse problem. Solving inverse problems is the process of computing the causal factors that give rise to a set of observations. These causal factors are typically either model parameters, the model itself, or the model output. There are, in general, two approaches for solving inverse problems: The variational approach, where a functional is minimized; and the Bayesian approach, where a posterior distribution is computed. We focus on the Bayesian approach, as we aim to resolve, in addition to a point estimate, the uncertainty associated with the estimate.

We use the notation $P_{x}$ for the probability distribution of the stochastic variable, x, and $p_{x}$ for the associated density function. $P_{x}\left(x_{i}\right)$ then denotes the probability of a specific value $x_{i}$.

The leak location is denoted by $c\in \{1,\ldots ,N_{p}\}$, the sensor observations by $y\in R^{N_{y}}$, the state is $x={\left(q^{T},h^{T}\right)}^{T}\in R^{N_{j}+N_{p}}$, the uncertain parameters are $\omega \in R^{N_{\omega }}$, $\omega ∼P_{\omega }$, the forward model is given by $F:R\times N_{\omega }\to R^{N_{j}+N_{p}}$, F(c,ω)=x, the observation operator by $H:R^{N_{j}+N_{p}}\to R^{N_{y}}$, H(x)=y, and the observation noise is $\eta \in R^{N_{y}}$, $\eta ∼P_{\eta }$. These quantities are related in the following way:

(6) $$\begin{array}{c} y=H(F(c,\omega ))+\eta . \end{array}$$

The forward model, *F*, is closely related to Equation (4). The output, F(c,ω)=x, is the solution to Equation (4) for a given *c* and ω. The uncertain parameters, ω, depend on the problem at hand, and can, for example, be the demand at each node or the pipe roughness in each pipe section. One would typically have some knowledge of the distribution, $P_{\omega }$, based on previous observations and calibration. The forward model is the function that maps the leak location and parameters to the solution of Equation (4). Hence, for larger WDNs, this can potentially be expensive to evaluate.

The distribution of interest is the posterior distribution of the leak location, i.e., the distribution over the leak location given the observations, $P_{c|y}$. Since *c* is a discrete variable, we have to compute the posterior probability of all possible values of *c*, $P_{c|y}\left(c_{k}|y\right)$, $c_{k}=1,\ldots ,N_{p}$. Using Bayes’ theorem for density functions, we obtain:

(7) $$\begin{array}{c} p_{c|y}\left(c_{k}|y\right)=\frac{p_{y|c}\left(y|c_{k}\right)p_{c}\left(c_{k}\right)}{p_{y}\left(y\right)}, \end{array}$$

where $p_{y|c}$ is referred to as the likelihood, $p_{c}$ is the prior, and $p_{y}$ the evidence. For leak detection, we often choose a uniform prior distribution. However, there are cases where prior information is known and incorporating it is highly beneficial. The evidence serves as a normalizing constant, which ensures that $p_{c|y}$ sums to one over all possible values of $c_{k}$. This is given by

(8) $$\begin{array}{c} p_{y}\left(y\right)=\sum_{k=1}^{N_{p}}p_{y|c}\left(y|c_{k}\right). \end{array}$$

The likelihood is not directly available; however, from Equation (6) we obtain:

(9) $$\begin{array}{c} p_{y|c,\omega }\left(y|c_{k},\omega \right)=p_{\eta }\left(y-H\left(F\left(c_{k},\omega \right)\right)\right), \end{array}$$

which implies that we can compute the likelihood by marginalizing over ω:

(10) $$\begin{array}{c} \begin{array}{cc} p_{y|c}\left(y|c_{k}\right) & =\int_{-\infty }^{-\infty }p_{y|c,\omega }\left(y|c_{k},\omega \right)p_{\omega |c}\left(\omega |c_{k}\right)d\omega \\ \; & =\int_{-\infty }^{-\infty }p_{y|c,\omega }\left(y|c_{k},\omega \right)p_{\omega }\left(\omega \right)d\omega \\ \; & =E_{\omega ∼P_{\omega }}\left[p_{y|c,\omega }\left(y|c_{k},\omega \right)\right]. \end{array} \end{array}$$

We assume that ω is independent of $c_{k}$.

As observations will arrive with time, we need to update the posterior when new observations become available, using the posterior from the previous observation time as the prior. We denote observations at time $t_{i}$ by $y_{i}$, which gives us the following posterior:

(11) $$\begin{array}{ccc} p_{c|y}\left(c_{k}|y_{i}\right) & = & \frac{p_{y|c}\left(y_{i}|c_{k}\right)p_{c|y}\left(c_{k}|y_{i-1}\right)}{p_{y}\left(y_{i}\right)}\\ & = & \frac{E_{\omega ∼P_{\omega }}\left[p_{y|c,\omega }\left(y_{i}|c_{k},\omega \right)\right]p_{c|y}\left(c_{k}|y_{i-1}\right)}{\sum_{j=1}^{N_{p}}E_{\omega ∼P_{\omega }}\left[p_{y|c,\omega }\left(y_{i}|c_{j},\omega \right)\right]}. \end{array}$$

The posterior distribution after observations at times $t_{0},t_{1},\ldots ,t_{N_{t}}$ is given by

(12) $$\begin{array}{c} p_{c|y}\left(c_{k}|y_{0:N_{t}}\right)=p_{c}\left(c_{k}\right)\prod_{i=0}^{N_{t}}\frac{E_{\omega ∼P_{\omega }}\left[p_{y|c,\omega }\left(y_{i}|c_{k},\omega \right)\right]}{\sum_{j=1}^{N_{p}}E_{\omega ∼P_{\omega }}\left[p_{y|c,\omega }\left(y_{i}|c_{j},\omega \right)\right]}. \end{array}$$

We can write down the expression $p_{c|y}\left(c_{k}|y_{0:N_{t}}\right)$ for all *k*, but it is not feasible to analytically solve it. Therefore, we need to make use of numerical approximations, such as Monte Carlo approaches. However, there are several computational challenges associated with this:

- $P_{\omega }$ is not necessarily known or it could be difficult to sample from;
- ω is, in general, high-dimensional. For example, when ω represents the stochastic demand in each node, then ω is $N_{j}$-dimensional. This makes the integral to be computed in Equation (10) high-dimensional, and it is thereby not feasible to compute the likelihood, $E_{\omega ∼P_{\omega }}\left[p_{c|y,\omega }\left(y_{i}|c_{k},\omega \right)\right]$, for all *k* in real time;
- $p_{c|y,\omega }\left(y_{i}|c_{k},\omega \right)$ can be expensive to evaluate as it requires solving Equation (4).

While several methods exist that address the computation of stochastic integrals, most of them have undesirable issues. For example, Gaussian processes (GPs) can be trained to compute the posterior distribution directly. However, with GPs, one is restricted to modeling multivariate Gaussian distributions. Furthermore, it is well known that kernel methods are, in general, not suitable for very high-dimensional cases [27]. An alternative to GPs is the polynomial chaos expansion (PCE), which allows for more complicated distributions than GPs. However, with PCE, one is typically even more restricted in dimensionality, as these methods suffer from the curse of dimensionality [28]. We will make use of deep learning, as it allows us to deal with arbitrary distributions and high-dimensional problems.

### 2.3. Supervised Wasserstein Autoencoder

The methodology chosen here to address the above-mentioned challenges is generative modeling. Particularly, we will focus our discussion on the use of the autoencoder set-up, whose details are explained in this subsection. A supervised Wasserstein autoencoder (SupWAE) represents a type of neural network that simultaneously achieves a problem dimensionality reduction and an approximation of the relevant distribution [24]. Before explaining the SupWAE, we briefly introduce the regular autoencoder (AE) and add the necessary components for leak detection.

#### 2.3.1. Autoencoders

A regular autoencoder (AE) is often used to identify an accurate low-dimensional representation of data [29]. This low-dimensional representation is then referred to as the latent state, which is an element of the latent space, while the original data are referred to as the high-fidelity state, belonging to the high-fidelity space.

An AE consists of two neural networks: An encoder, $\phi_{\mathrm{enc}}$, that sparsifies (i.e., reduces the dimensionality of) the data, and a decoder, $\phi_{\mathrm{dec}}$, that reconstructs the data:

(13) $$\begin{array}{c} \phi_{\mathrm{enc}}\left(x\right)=z,\;\phi_{\mathrm{dec}}\left(z\right)=\tilde{x},\;\phi_{\mathrm{dec}}\left(\phi_{\mathrm{enc}}\left(x\right)\right)=\tilde{x}\approx x. \end{array}$$

Considering a training set, ${\left\{x_{i}\right\}}_{i=1}^{N}∼P_{x}\left(x\right)$, AEs are trained by minimizing the mean squared error (MSE) with a weight regularization:

(14) $$\begin{array}{c} L_{\mathrm{AE}}\left(\phi_{\mathrm{enc}},\phi_{\mathrm{dec}}\right)=\frac{1}{N}\sum_{zi=1}^{N}{\left(x_{i}-\phi_{\mathrm{dec}}\left(\phi_{\mathrm{enc}}\left(x_{i}\right)\right)\right)}^{2}+\alpha R\left(\phi_{\mathrm{enc}},\phi_{\mathrm{dec}}\right) \end{array}$$

$L_{\mathrm{AE}}$ is minimized with respect to the weights of $\phi_{\mathrm{enc}}$ and $\phi_{\mathrm{dec}}$, typically using stochastic gradient-descent-type algorithms. The term $R(\phi_{\mathrm{enc}},\phi_{\mathrm{dec}})$ is chosen to be the $l^{2}$ norm of the weights. This is referred to as the weight decay within machine learning; α determines how much we regularize the weights. The purpose of this regularization is to avoid overfitting to the training data.

#### 2.3.2. Wasserstein Autoencoders

While AEs provide a framework for computing latent representations of data, they lack certain properties that are of interest when computations in the latent space are necessary. Specifically, small perturbations in the latent space should also result in small perturbations in the high-fidelity space. Moreover, it should be possible to sample from the latent space. By using Wasserstein AEs (WAEs) [24], we can also obtain these properties.

We introduce a prior distribution to the latent space, $P_{z}\left(z\right)$. While the encoder and decoder remain deterministic mappings, they define the conditional distributions $P_{\mathrm{enc}}\left(z|x\right)$ and $P_{\mathrm{dec}}\left(x|z\right)$, respectively, using the densities:

(15) $$\begin{array}{c} \begin{array}{c} p_{z}\left(z\right)=\int_{x}p\left(z|x\right)p_{x}\left(x\right)dx\approx \int_{x}p_{\mathrm{enc}}\left(z|x\right)p_{x}\left(x\right)dx=p_{\mathrm{enc}}\left(z\right), \end{array} \end{array}$$

(16) $$\begin{array}{c} \begin{array}{c} p_{x}\left(x\right)=\int_{z}p\left(x|z\right)p_{z}\left(z\right)dz\approx \int_{z}p_{\mathrm{dec}}\left(x|z\right)p_{z}\left(z\right)dz=p_{\mathrm{dec}}\left(x\right). \end{array} \end{array}$$

Here, a sample from $P_{\mathrm{enc}}\left(z|x\right)$ is computed using the encoder, $z=\phi_{\mathrm{enc}}\left(x\right)$, and similarly a sample from $P_{\mathrm{dec}}\left(x|z\right)$ is obtained using the decoder, $x=\phi_{\mathrm{dec}}\left(z\right)$. The goal is to ensure that the encoder approximates the latent prior distribution, $P_{z}\left(z\right)\approx P_{\mathrm{enc}}\left(z\right)$, and the decoder approximates the high-fidelity prior distribution, $P_{x}\left(x\right)\approx P_{\mathrm{dec}}\left(x\right)$. This is achieved by simultaneously minimizing the reconstruction error and a divergence, *D*, between $P_{z}\left(z\right)$ and $P_{\mathrm{enc}}\left(z\right)$, which measures the similarity of the two distributions:

(17) $$\begin{array}{c} L_{\mathrm{WAE}}\left(\phi_{\mathrm{enc}},\phi_{\mathrm{dec}}\right)=\underset{\mathrm{reconstruction}}{\underset{︸}{\frac{1}{N}\sum_{i=1}^{N}{\left(x_{i}-\phi_{\mathrm{dec}}\left(\phi_{\mathrm{enc}}\left(x_{i}\right)\right)\right)}^{2}}}+\lambda \underset{\mathrm{divergence}}{\underset{︸}{D(P_{z}\left(z\right),P_{\mathrm{enc}}\left(z\right))}}+\alpha \underset{\mathrm{regularization}}{\underset{︸}{R(\phi_{\mathrm{enc}},\phi_{\mathrm{dec}})}}. \end{array}$$

Here, λ is another regularization parameter to be determined through hyperparameter tuning. In this paper, we choose the maximum mean discrepancy (MMD) with a multiquadratics kernel for the divergence, which gives us the WAE-MMD. In [30], it was argued that this is an accurate choice when $P_{z}\left(z\right)$ is the normal distribution, see [24]. In summary, we obtain:

$$\begin{array}{ccc} L_{\mathrm{WAE}}\left(\phi_{\mathrm{enc}},\phi_{\mathrm{dec}}\right) & = & \frac{1}{N}\sum_{i=1}^{N}{\left(x_{i}-\phi_{\mathrm{dec}}\left(\phi_{\mathrm{enc}}\left(x_{i}\right)\right)\right)}^{2}+\lambda \mathrm{MMD}\left(\phi_{\mathrm{enc}},{\left\{z_{i}\right\}}_{i=1}^{N}\right)\\ & + & \alpha R(\phi_{\mathrm{enc}},\phi_{\mathrm{dec}}), \end{array}$$

where $z_{i}∼P_{z}\left(z\right),$

$$\begin{array}{ccc} \mathrm{MMD}\left(\phi_{\mathrm{enc}},{\left\{z_{i}\right\}}_{i=1}^{N}\right) & = & \frac{\lambda }{N(N-1)}\sum_{l\neq j}^{N}\left[k\left(z_{l},z_{j}\right)+k\left(\phi_{\mathrm{enc}}\left(x_{l}\right),\phi_{\mathrm{enc}}\left(x_{j}\right)\right)\right]\\ & + & \frac{2\lambda }{N^{2}}\sum_{l,j}^{N}k(z_{l},\phi_{\mathrm{enc}}\left(x_{j}\right), \end{array}$$

and

(18) $$\begin{array}{c} k\left(z_{l},z_{j}\right)=\frac{C}{C+\left|\right|z_{l}-z_{j}{\left|\right|}_{2}^{2}}. \end{array}$$

After training, it is possible to sample from the chosen latent prior distribution, $p_{z}\left(z\right)$, pass it through the decoder, $\phi_{\mathrm{dec}}$, and obtain a high-fidelity sample, $\phi_{\mathrm{dec}}\left(z\right)=x$.

**Figure 1:**
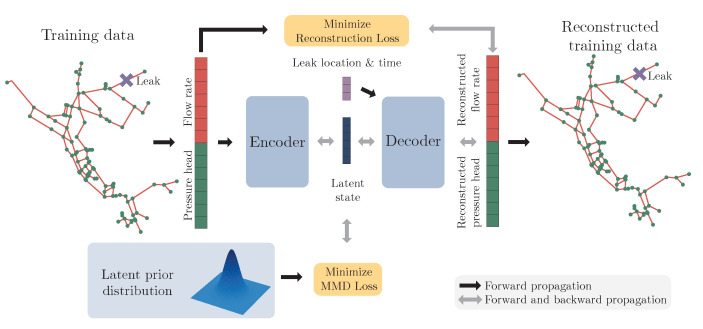
Illustration of a Wasserstein AE for reconstruction of a water distribution network.

To obtain the supervised version of the WAE, we introduce *c* as an extra input to the decoder, so $\phi_{\mathrm{enc}}\left(\cdot \right):=\phi_{\mathrm{enc}}\left(z,c\right)$. In this way, the decoder models the conditional probability density function, $p_{x|c}\left(x|c\right)$,

(19) $$\begin{array}{c} \begin{array}{cc} p_{x|c}\left(x|c\right)\; & =\int_{z}p_{x|c,z}\left(x|c,z\right)p_{z}\left(z\right)dz\\ & \approx \int_{z}p_{\mathrm{dec}}\left(x|c,z\right)p_{z}\left(z\right)dz=\int_{z}\phi_{\mathrm{dec}}\left(z,c\right)p_{z}\left(z\right)dz=p_{\mathrm{dec}}\left(x|c\right), \end{array} \end{array}$$

so that *c* is considered a known condition. During training, the WAE sees pairs (x,c) and uses this information to learn the conditional distribution. The condition *c* determines the class, while the latent variable, z, determines the style. This separation will be crucial for the proposed framework. A visualization of the supervised WAE-MMD is shown in Figure 1.

### 2.4. Neural Network Architectures

Different kinds of neural network architecture can be incorporated into the WAEs framework. Therefore, one should choose the architecture that performs optimally for the data type. In this paper, we showcase the performance using two different kinds of architecture—residual neural networks (ResNet) [31] and transformers.

#### 2.4.1. Residual Neural Networks

A residual neural network is a neural network that consists of residual layers. A residual layer is defined as having a skip connection bypassing the normal layer. That is, a residual layer is given by

(20) $$\begin{array}{c} x^{i+1}=F^{i}\left(x^{i}\right)+x^{i}, \end{array}$$

where $x^{i}$ is the output of layer *i* and $F^{i}$ is the *i*th layer. $F^{i}$ typically consists of a linear layer, followed by an activation function, and then another linear layer. The linear layers can be either dense or convolutional.

The advantage of using ResNets is that the problem of vanishing gradients is not very apparent, which makes them easier to train [31].

#### 2.4.2. Transformers

Transformers are another type of neural network architecture, introduced in 2017 [32], that became the default choice for natural language processing. Since then, transformers have outperformed the state-of-the-art methodologies in areas such as image recognition [33], protein structure prediction [34], and time series forecasting [35]. While these applications seem very different from leak detection in water distribution networks, they share some features that the transformer architecture addresses well.

Transformers treat the data as if it were a fully connected graph. The multi-head attention models how information from one node or pipe section should be aggregated to another node or pipe section. In this way, the neural networks learn which connections should be strengthened and which should be weakened. Hence, the transformer can efficiently learn long-range relations between nodes and pipe sections. This is in sharp contrast to graph neural networks, where many layers are necessary to capture such long-range relations.

For a more technical presentation of the transformer architecture and the attention mechanism, we refer to Appendix A.

## 3. Proposed Framework

The aim of the proposed framework is to overcome the challenges related to solving Bayesian inverse problems, as described in Section 2.2, while maintaining a high accuracy when computing the posterior over the parameters of interest. The framework employed here is an extension of the one presented in [17].

We will use a generative neural network as a stochastic digital twin of the WDN. This can be used to sample pressure heads and flow rates of the entire WDN for a given leak location and time. By modeling the pressure heads and flow rates as distributions conditioned on leak location and time, instead of a deterministic output, the uncertainty due to the stochastic parameters is included in the model output. A generative neural network is a suitable choice for this, as this enables us to sample from the distribution of pressure heads and flow rates.

We will specifically make use of the generative properties of the decoder of the supervised WAE-MMD, whereas the encoder is discarded after training. The decoder is trained to approximate the state, when given the random noise, leak location, and the time of day.It then replaces the forward model, and the latent vector, z, replaces the uncertain parameters; in our case, the demand. Using the same approach as in Section 2.2, we can rewrite the posterior for given observations, as follows:

(21) $$\begin{array}{c} p_{c|y}\left(c_{k}|y_{i}\right)=\frac{p_{c}\left(c_{k}\right)\int_{z}p_{y|c,z}\left(y_{i}|c_{k},z\right)p_{z}\left(z\right)\;dz}{\sum_{j=1}^{N_{p}}\int_{z}p_{y|c,z}\left(y_{i}|c_{j},z\right)p_{z}\left(z\right)\;dz}=\frac{p_{c}\left(c_{k}\right)E_{z∼P_{z}}\left[p_{y|c,z}\left(y_{i}|c_{k},z\right)\right]}{\sum_{j=1}^{N_{p}}E_{z∼P_{z}}\left[p_{y|c,z}\left(y_{i}|c_{j},z\right)\right]}, \end{array}$$

where the likelihood is computed by

(22) $$\begin{array}{c} p_{y|c,z}\left(y_{i}|c_{k},z\right)=p_{\eta }\left(y_{i}-H\left(\phi_{\mathrm{dec}}\left(z,c_{k}\right)\right)\right). \end{array}$$

As in Equations (11) and (12), we can use the posterior from time $t_{i-1}$ as the prior for the posterior at time $t_{i}$. This gives us the resulting posterior for a series of $N_{t}$ observations:

(23) $$\begin{array}{c} p_{c|y}\left(c_{k}|y_{0:N_{t}}\right)=p_{c}\left(c_{k}\right)\prod_{i=0}^{N_{t}}\frac{E_{z∼P_{z}}\left[p_{y|c,z}\left(y_{i}|c_{k},z\right)\right]}{\sum_{j=1}^{N_{p}}E_{z∼P_{z}}\left[p_{y|c,z}\left(y_{i}|c_{j},z\right)\right]}. \end{array}$$

At time $t_{0}$, we simply use a uniform prior.

As an addition to the described setup, we add the timestamp as an additional input to the decoder. This gives us the following description:

(24) $$\begin{array}{c} \phi_{\mathrm{dec}}\left(z,c,t_{i}\right)=x_{i}\left(c,\omega \right)={\left(q_{i}^{T}\left(c,\omega \right),h_{i}^{T}\left(c,\omega \right)\right)}^{T}. \end{array}$$

With this formulation the decoder disentangles the temporal information from the rest. Hence, the time dependency of the stochastic demand is explicitly modeled in the decoder.

The proposed framework essentially resolves the challenges described in Section 2.2:

- $P_{\omega }$ is replaced by the latent prior, $P_{z}$, which is known, as we used it during training of the WAE-MMD;
- z is of a much lower dimension than ω, which makes the evaluation of the integral in Equation (10) fast and thereby enables real-time computation of the likelihood, $E_{z∼P_{z}}\left[p_{y|c,z}\left(y_{i}|c_{k},z\right)\right]$;
- The likelihood, $p_{y|c_{k},z}$, is computed using a forward propagation of the decoder, instead of solving an expensive forward model.

**Figure 2:**
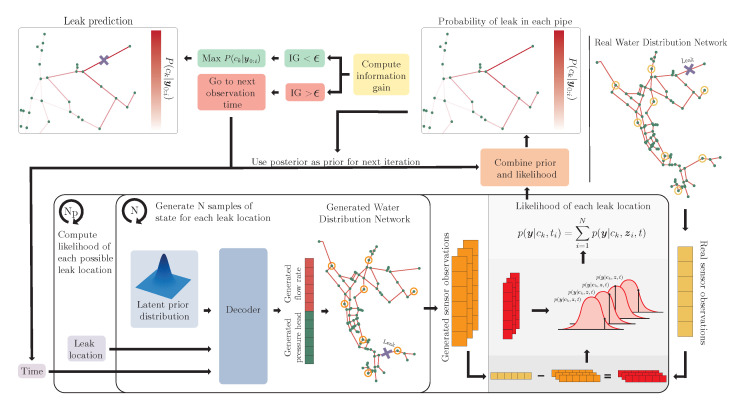
Illustration of the online computation of the posterior distribution, $p(c_{k}|y_{0:i})$, $k=1,\ldots ,N_{p}$.

While the costs are drastically reduced in the leak detection stage, the training stage is now (potentially) expensive to compute. These two stages are referred to as the online stage, in which the trained supervised WAE-MMD is used to solve the leak localization problem for given observations, and the offline stage, in which we generate training data and train the supervised WAE-MMD. These two stages are outlined in Algorithms 1 and 2, respectively. Furthermore, the online stage is visualized in Figure 2.
**Algorithm 1:** Offline stage   **Input**: $N_{\mathrm{train}}$, training hyperparameters, WAE-MMD architecture1Generate training samples, ${\left\{\left(x_{i},c_{i}\right)\right\}}_{i=1}^{N_{\mathrm{train}}}$, by solving the forward problem (see Section 2.1);2Train the supervised WAE-MMD (see Section 2.3);
    **Output**: $\phi_{\mathrm{dec}}$

**Algorithm 2:** Online stage

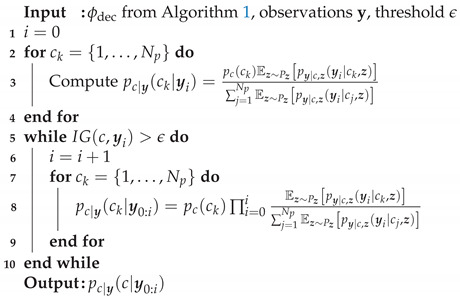



### 3.1. Stopping Criterion in the Online Stage

With every new set of observations, $y_{i}$, we receive additional information about the system at hand. However, at some point, new observations no longer contribute to the accuracy of the posterior. In other words, the posterior density should converge:

(25) $$\begin{array}{c} p_{c|y}\left(c_{k}|y_{0:N_{t}}\right)\to p_{c|y}\left(c_{k}|y\right),\;\mathrm{for}\;N_{t}\to \infty . \end{array}$$

This implies that the algorithm should be stopped when there is no significant change to the posterior. For this reason, we introduce the information gain of *c*, $IG(c,y_{i})$, obtained from additional observations, as the stopping criterion. The information gain is defined by the KL divergence between the posterior at time $t_{i}$ and time $t_{i-1}$, which measures how much the posterior distribution changed with new observations:

(26) $$\begin{array}{c} \begin{array}{cc} IG(c,y_{i}) & =D_{\mathrm{KL}}(P_{c|y}\left(c_{k}|y_{0:i}\right)\left|\right|P_{c|y}\left(c_{k}|y_{0:i-1}\right))\\ \; & =-\sum_{k=1}^{N_{p}}p_{c|y}\left(c_{k}|y_{0:i}\right)\log\left(\frac{p_{c|y}\left(c_{k}|y_{0:i}\right)}{p_{c|y}\left(c_{k}|y_{0:i-1}\right)}\right). \end{array} \end{array}$$

We terminate the computations when the information gain is below a threshold, ϵ.

### 3.2. Estimating Uncertainty

In order to decide whether a leak location prediction is trustworthy, we can compute the entropy of the posterior:

(27) $$\begin{array}{c} H\left(C\right)=-\sum_{i=1}^{N_{p}}p_{c}\left(c_{i}|y_{0:1}\right)\log\left(p_{c}\left(c_{i}|y_{0:1}\right)\right). \end{array}$$

This tells us how much information or uncertainty we have in the posterior distribution. A low entropy means a sufficient amount of information, i.e., low uncertainty, and a high entropy signifies the opposite. Therefore, we can use the entropy as a measure of how much we can trust our prediction. That is, when there are insufficient observations to make a trustworthy prediction, the entropy will be higher and thereby indicate that more observations are needed. Hence, when the truth is unknown, we can still assess whether the prediction is accurate or not.

This is a crucial step in applying the methodology in practice, as it provides the necessary information to act on a certain prediction. If the entropy is high, this tells us that we need more observations in order to provide a trustworthy prediction.

### 3.3. Model Architectures

**Figure A2:**
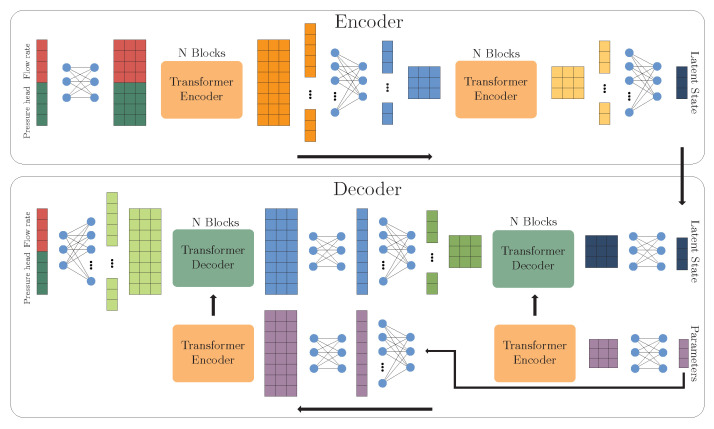
Illustration of the encoder and decoder architectures.

As mentioned in Section 2, the WAE and the proposed framework do not rely on only one neural network architecture. In this work, we make use of two different types of neural network architecture, to demonstrate that the framework can be based on more than one possible choice. Moreover, we will see that each choice has a superior performance for a certain test case. Specifically, we use transformers and dense ResNets in the experiments that follow. For a detailed breakdown of the model architectures, see Appendix B, and for a visualization of the transformer network, see Figure A2. For the transformers, both the encoder and the decoder make use of a combination of dense neural networks, transformer encoders, and transformer decoders. With this architecture, the network structure of the data is modeled using attention mechanisms. For the dense ResNet, the encoder and decoder consist of a series of residual layers with dimension reduction and increasing layers, respectively, in between.

## 4. Results

In this section, we show the performance of the proposed framework in three test cases. We assessed the framework’s performance with respect to the topological distance and accuracy (The code used for generating the data and the corresponding results can be found on GitHub, see https://github.com/nmucke/DT-for-WDN-leak-localization.git (accessed on 13 June 2023)). We compared the performance of two distinct architectures, a dense ResNet and a transformer architecture. In both cases, the neural networks were similar to the encoder network, but with a softmax activation function at the output.

For comparison, we also computed the leak location using a conventional classification neural network, which was trained to classify a leak location based on sensor observations at a given time. The classification network was trained on the same data as the autoencoders. It is worth noting that, with this method, it is necessary to train a model for each sensor configuration. This is in contrast to the proposed framework, where a single model can handle all possible sensor configurations. For more details on the classification model, see Appendix D.

### 4.1. Test Cases

**Figure 3:**
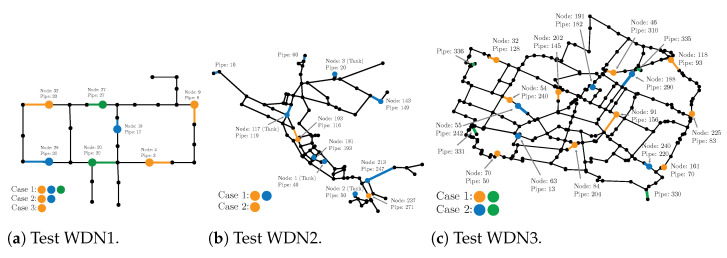
Network topologies and the sensor locations for the three test cases.

All test cases were defined in a similar manner, however, with some variations. Figure 3 shows the three test WDN topologies, together with the sensor locations. The first one is typically referred to as a Hanoi network; the second is often referred to as Net3 (not to be confused with the numbering of the cases in this paper), and the third is known as Modena.

We placed sensors in various nodes in the WDN. Each sensor measured the pressure head value in the node and the flow rate in a neighboring pipe section. We did not use pressure head sensors at the water sources, as such information does not make sense in many cases, e.g., for lakes and rivers. However, we placed flow rate sensors in pipes connected to those sources, in order to mimic a real-world scenario, where the inflow into the network is measured. For test case 1, we show results for three configurations, each with a varying number of sensors. For test cases 2 and 3, we show results for two sensor configurations. Note that, since the generative model outputs the full state, consisting of pressure heads and flow rate, the different sensor configurations only changed the observation operator in the online stage; that is, only *H* in (22) was varied with the different sensor configurations.Hence, a single training stage was performed, and the resulting generative model worked under multiple sensors settings.

We also added noise to the sensor readings, to mimic a real-world scenario where the sensors would not be perfect. In all cases and for all pressure head and flow rate observations, the noise was sampled from a normal distribution at each time step and added to the observations. The noise at each sensor was independent of the other sensors. The normal distribution had a mean of zero and a standard deviation corresponding to a percentage of the observed value. We show results for various noise percentages in all test cases, to analyze the performance of the algorithm in various settings. In Table 1, we show the specific parameter and noise settings for the three test cases.

In all test cases, the data were generated by varying the pipe section in which the leak was present. The leak size was also varied by varying the leak area between $0.002\;[m^{2}]$ ($20\;[{\mathrm{cm}}^{2}]$) and $0.004\;[m^{2}]$ ($40\;[{\mathrm{cm}}^{2}]$). The test cases were simulated for 24 h, with values recorded every hour.

**Figure 4:**
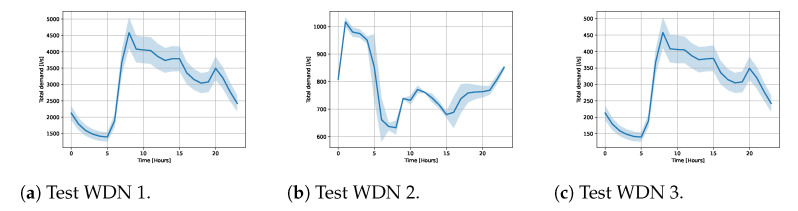
Total demand with standard deviation.

**Figure 5:**
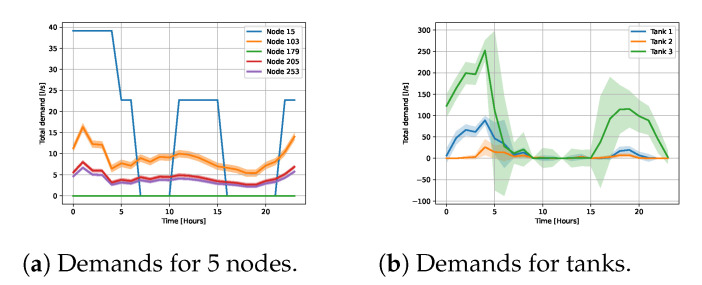
Demand patterns for the test WDN 2.

All the demands followed a temporal pattern, i.e., in each node at every time of the day, the demand had a base value. In order to mimic the stochasticity of the demand, we added noise to the base values. The total demands in each network are shown in Figure 4 and examples of demand patterns for individual nodes are shown in Figure 5.

We used two metrics to assess the performance of the framework: the average topological distance (ATD), and the accuracy. The accuracy is the fraction of correctly predicted leak locations. The topological distance is the distance of the shortest route from the predicted leak location to the true leak location. The ATD was then computed by taking the average of the many different solutions of the inverse problems for varying leak locations, sizes, demands, and sensor noises.

Furthermore, it is important to emphasize that the models were trained on a dataset that was distinct from the test dataset used in assessing the performance. The Epanet .inp files can be found in the GitHub repository.

### 4.2. Training of the WAEs

The WAEs were trained on simulated data. Training data were generated by simulating according to the settings described above. The leak locations were sampled from a multinomial distribution, with an equal probability assigned to each pipe section. The leak size was sampled uniformly from the interval $\left[0.002\;m^{2},0.004\;m^{2}\right]$. Furthermore, the demand noise was sampled at each time instance from a normal distribution with a mean 0 and a variance equal to 10% of the base value in test cases 1 and 3 and 5% in test case 2.

The hyperparameter settings for the WAEs are described in Appendix C.

### 4.3. Test WDN1: Hanoi Network

For test WDN1, we considered three different sensor configurations (see Figure 3a), each with five different sensor noise levels. All nodes are given the same demand pattern, but with varying base values in each node. Noise was added to the total demand, which was then distributed to all nodes according to the relative base value. Noise was added to each node independently of the other nodes. See Figure 4a for the total demand time series with the standard deviation.

**Figure 6:**
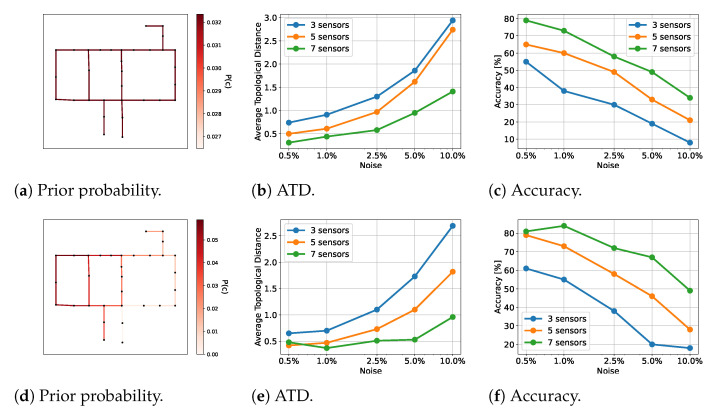
Results for WDN1 for the given priors. The first row shows results for the prior in (**a**) and the second row shows results for the prior in (**d**). The number of sensors counted is the number of flow rate sensors. Note that there are fewer pressure head sensors.

We tested the framework with both a prior distribution of the leak locations and without any prior knowledge. The prior distribution is shown in Figure 6d.

We made use of a transformer architecture here. The results are shown in Figure 6. The likelihood was computed with 30,000 samples. We tested using 50 different leak locations.

As expected, the ATD increased and the accuracy decreased with increasing noise and a decreasing number of sensors. The ATD went from approximately 0.25 to approximately 3.0 from the best to the worst case, and the accuracy diminished from approximately 79% to approximately 9% when no prior was available. When a prior was present, the ATD ranged from approximately 0.4 to approximately 2.7, and the accuracy ranged from approximately 81% to approximately 19%, which was a drastic reduction.

### 4.4. Test WDN2

For test WDN2, we considered two different sensor configurations (see Figure 3b), each with five different sensor noise levels. The total demand is shown Figure 4b. There was a varying demand pattern for each node. Some nodes mimicked households, other nodes had no demand, and some had demands mimicking factories. Noise was added to each node independently of the other nodes. In Figure 5a, examples of the demand patterns are presented. Furthermore, there were water tanks present, which served as stabilizers for the WDN. Hence, their demand varied according to the total demand. In Figure 5b, examples of three tanks are shown.

**Figure 7:**
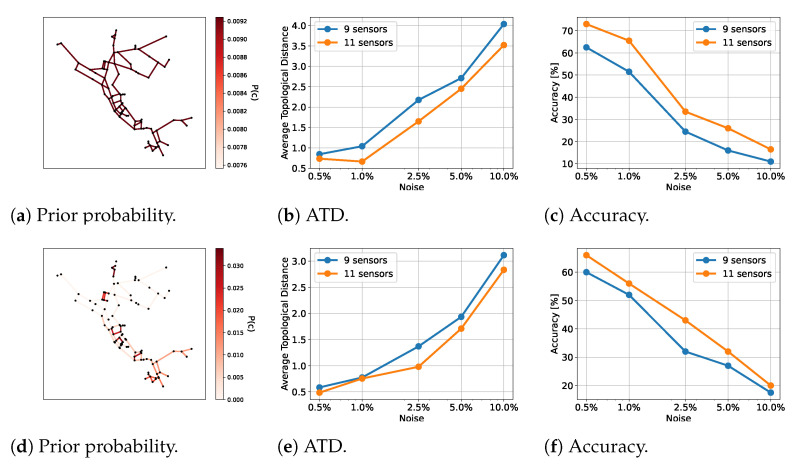
Results for WDN2 for the given priors. The first row shows the results for the prior in (**a**), and the second row shows the results for the prior in (**d**). The number of sensors counted is the number of flow rate sensors. Note that there are fewer pressure head sensors.

As for test WDN1, we worked with a prior distribution of the leak locations and without any prior knowledge. The prior distribution is shown in Figure 7d. We made use of a transformer architecture. The results are shown in Figure 7. The likelihood was computed with 30,000 samples. We tested with 200 different leak locations.

The results were very similar to the results for WDN1, which suggests that the framework scales well to more complicated settings.

### 4.5. Test WDN3: Modena

For test WDN3, we again considered two different sensor configurations (see Figure 3c), each with five different sensor noise levels. The demand patterns were modeled in the same way as for WDN1, but with a different total demand. See Figure 4c for the total demand time series.

Uniquely to this test case, we made use of a different standard deviation for the likelihood computation than for the noise added to the observations. Specifically, we used a standard deviation of 5%, no matter the artificial noise added to the observations. This is more reminiscent of a real-world scenario, where the true sensor noise would be unknown.

**Figure 8:**
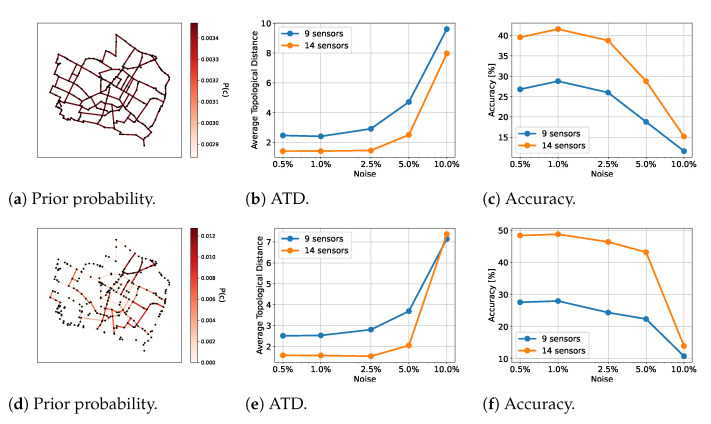
Results for WDN3 for the given priors. The first row shows the results for the prior in (**a**), and the second row shows the results for the prior in (**d**). The number of sensors counted is the number of flow rate sensors. Note that there are fewer pressure head sensors.

As for test cases WDN1 and WDN2, we worked with a prior distribution of the leak locations and without any prior knowledge. The prior distribution is shown in Figure 8d. Here, we made use of the ResNet architecture. The results are shown in Figure 8. The likelihood was computed with 50,000 samples. We tested with 250 different leak locations.

As for the other two test cases, we saw a better performance when we made use of prior information. Furthermore, increasing the number of sensors also gave better results.

We saw a decrease in accuracy and an increase in ATD when the noise level was increased. However, in contrast to the other test cases, the performance was relative constant across noise levels, until 5% and 10%, where the accuracy and ATD worsened. This suggests that our method is stable until a certain threshold, where more information is needed to say something definite about the leak location. This is not a surprise, as the WDN was significantly larger than in the two other test cases and therefore might require additional observations when there is a lot of noise present. In such cases it is of great value that our method also quantifies the uncertainty associated with the prediction. Hence, the user will know when there is insufficient information to come to a strong conclusion.

### 4.6. Comparison with the Baseline

In Table 2, we show a comparison of the results computed with our framework and the baseline classifier. Clearly, the proposed framework outperformed the classifier for both WDN1 and WDN2 with both the ResNet and the transformer architectures. Furthermore, the transformer network gave the best results, suggesting that the transformer architecture is a suitable choice for small to medium-sized WDNs.

For WDN3, the results were different. The ResNet architecture with the new framework gave the best results, while the transformer architecture did not perform very well. However, with the classifier, the transformer architecture performed the best, suggesting that the transformer architecture may also be a satisfactory choice in that setting.

### 4.7. Posterior Distribution Entropy

To evaluate the entropy computations, we considered three different entropy functions, i.e., the mean entropy of the posterior for all incorrect predictions, $E[H_{\mathrm{wrong}}\left(C\right)]$, the mean entropy of the posterior for all correct predictions $E[H_{\mathrm{correct}}\left(C\right)]$, and the ratio between the two $E\left[H_{\mathrm{wrong}}\left(C\right)\right]/E\left[H_{\mathrm{correct}}\left(C\right)\right]$. This ratio informs us about the relative size of the entropy of an incorrect prediction compared to a correct prediction. Ideally, this ratio should be large, as this is an indication that the framework provides high uncertainty for incorrect predictions and a low uncertainty for correct predictions.

**Figure 9:**
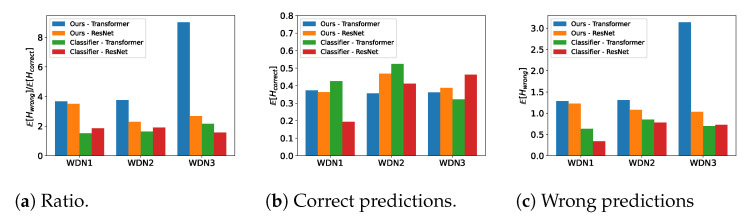
Entropy of the estimated posterior distributions. (**a**) Shows the ratio between the mean entropy of the posterior for a wrong prediction and a correct prediction. (**b**) Shows the mean entropy of the posterior distribution for correct predictions. (**c**) Shows the mean entropy of the posterior distribution for wrong predictions.

In Figure 9, it is clear that, compared with the classifier, the new framework showed consistently higher entropy ratios. Thus, the framework provided more accurate uncertainty estimates compared to the classifier. In particular, for the transformer neural network in the new approach, the entropy ratio was high, because the entropy for incorrect predictions was high. In summary, if the ATDs and accuracy were not high, this would be reflected in the entropy. Therefore, we know a posteriori, and accurately, when predictions computed with the new framework are trustworthy. This is an important feature and one that is not easily attained.

## 5. Conclusions

We presented a framework for computing leakage locations using Bayesian inference and generative deep learning. A Bayesian approach was used to formulate the leak localization in a probabilistic manner. A generative neural network was trained to approximate the distribution of pressure heads and flow rates given a leak location and time using the WAE framework. To use the generative neural network for leak localization, the Bayesian problem was reformulated to a latent Bayesian inference problem. The approach was showcased in three test cases and showed a superior performance compared to a classification approach.

A Bayesian approach to leak localization offers two distinct advantages compared to non-probabilistic methods. First, it automatically gives the uncertainty of the prediction, in the shape of the posterior distribution. Second, it allows one to incorporate prior knowledge. However, it comes at a cost to the computation time.

The generative deep learning is trained in an offline stage on simulated data. After training, it then serves as a fast to evaluate surrogate model. In other words, the Bayesian inverse problem can be solved efficiently and without sacrificing accuracy when the neural network is trained properly.

We showed that one can obtain good quality performance using different architectures. We specifically showcased results using transformers and dense ResNets. The transformer architecture performed best in the two first test cases, while the ResNet performed best in the last test case. Therefore, one should investigate which neural network architecture to use in advance. However, the fact that the framework works with various architectures means that new state-of-the-art architectures can easily be incorporated.

For all test cases considered, the new framework outperformed the classification approach. Furthermore, the flexibility of the framework was shown through varying the amount of noise in the nodal demand and sensor measurements. Even when the true noise level was unknown, it was shown in test case 3 that the method performed well. Furthermore, the (positive) impact of prior knowledge of the leak location on the accuracy was detailed.

The framework not only provides leak location estimates, but also estimates of the uncertainty in the estimates. This is achieved by means of the entropy of the posterior distribution. We showed that the entropy computed with the new framework was significantly more informative than for the classification approach.

This paper thus showed the potential of using generative deep learning as a stochastic digital twin for water distribution networks, as it was seamlessly integrated with Bayesian inversion schemes.

There are many opportunities for further study. The computation of the posterior could be sped up with more efficient integration methods than Monte Carlo simulation. The physics of the problem could be incorporated into the training of the neural network, to ensure conservation of important quantities such as mass and momentum.

## Figures and Tables

**Table 1:** Settings for the three test cases.

	WDN 1	WDN 2	WDN 3
Num. pipes	34	119	317
Num. junctions	31	97	272
Demand noise	10%	5%	10%

**Table 2:** Comparison with the baseline. The ATD and accuracy were computed for all noise and sensors cases, both with a prior and without a prior, and then averaged. Our framework is denoted by “Bayes”, followed by the neural networks utilized. The vertical arrows denote the desired direction of the metric. The best results are highlighted in boldface.

	WDN1		WDN2		WDN3	
	ATD ↓	Acc ↑	ATD ↓	Acc ↑	ATD ↓	Acc ↑
Classifier-ResNet	2.94	26.5	5.81	13.25	10.01	7.85
Classifier-Transformer	2.06	31.67	3.60	21.5	4.88	27.92
Bayes-WAE, ResNet	1.23	45.70	2.91	25.03	**3.49**	**29.50**
Bayes-WAE, Transformer	**1.07**	**50.00**	**1.71**	**39.28**	10.38	4.54

**Figure A1:**
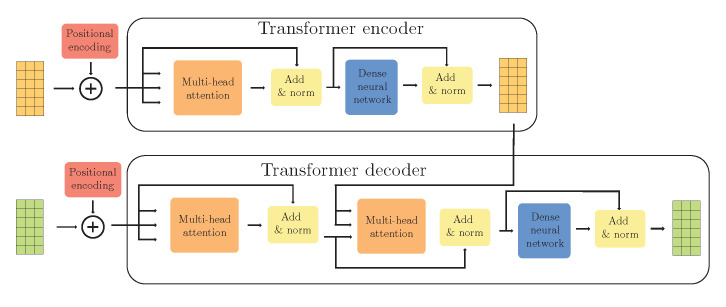
Transformer encoder and decoder architecture.

## Data Availability

The code for generating the synthetic data used in the paper can be found in the GitHub repository: https://github.com/nmucke/DT-for-WDN-leak-localization.git (accessed on 13 June 2023)).

## Appendix A. Attention and Transformers

We will highlight the relevant features of the transformer architecture used in this work. The key to the transformer architecture is the so-called attention mechanism. The scaled dot-product attention is the method of choice here. For a matrix, $X\in R^{k\times d}$, the scaled dot-product attention is computed by

(A1a) $$\begin{array}{c} K=F_{k}\left(X\right)\in R^{k\times d},\;Q=F_{q}\left(X\right)\in R^{k\times d},\;V=F_{v}\left(X\right)\in R^{k\times d}, \end{array}$$

(A1b) $$\begin{array}{c} Attention\left(Q,K,V\right)=softmax\left(\frac{QK^{T}}{\sqrt{d}}\right)V, \end{array}$$

where *K* refers to the keys, *Q* the queries, and *V* the values. $F_{k}$, $F_{q}$, and $F_{v}$ are typically shallow neural networks to be fitted during training. *k* is the context length and *d* is the embedding dimension. An attention layer typically consists of so-called multiple attention “heads”. Each head is of the same structure but consists of different functions, $F_{k}$, $F_{q}$, and $F_{v}$, resulting in multiple attention maps, $C_{i}$, that are concatenated along the $d^{th}$ dimension.

By connecting the attention layer to a residual connection, a normalization layer, a dense neural network, another residual connection, and another normalization, we have the transformer encoder module. See Figure A1 for a visualization.

With matrix *X*, a sequence of *k* elements, with each element being a vector of size *d*, the attention mechanism computes how much each element in the sequence should attend to every other element. In the context of natural language processing, the attention mechanism computes how much every word in a sequence is influenced by any other word in the sequence. A slightly more suitable interpretation in the present application is to consider *X* a graph with *k* nodes and *d* features in each node. The attention mechanism then computes how strong the connection should be between each pair of nodes.

In Equation (A1), the attention described is referred to as self-attention, referring to the fact that all elements of *X* only attend to other elements in *X*. Similarly, one defines cross-attention using the attention map from another source as keys and values. By combining this with self-attention, normalization, and a dense neural network, we have the decoder transformer module, see also Figure A1 for a visualization.

The transformer encoder and decoder networks are not aware of the relative positions of the individual nodes. Therefore, positional encoding is added to the features before passing them through the transformers.

## Appendix B. Model Architecture

### Appendix B.1. Dense ResNet

The encoder consists of a series of ResNet layers, followed by dense layers that reduce the dimensionality. Similarly, the decoder consists of ResNet layers, followed by dense layers that increase the dimensionality. The input layer of the encoder takes a vector with the flow rates and pressure heads concatenated and outputs the latent state. The decoder takes the latent state and the parameters (leak location and time) concatenated and outputs the full order state.

### Appendix B.2. Transformer

For the encoder, the data are first passed through a dense layer, in order to increase the embedding dimension. Then, they are passed through several transformer encoder layers. The transformer layers model interactions between the nodes and edges of the network. The resulting attention maps are reshaped into a vector and passed through dense layers, in order to decrease the dimensionality. Lastly, the reduced representation of the network is passed through transformer encoder layers, in order to model the relations between the latent features. The decoder follows a similar structure, the difference being the inclusion of the parameters and transformer decoder layers instead of encoder layers. The parameters are passed through two different sets of transformer encoder layers. The output of the first set of transformer encoder layers is used as input to the cross-attention in the latent transformer decoder layers. The output of the second transformer encoder layer is passed to the full state space decoder transformer layers. For a visualization of the architectures, see Figure A2.

## Appendix C. WAE Training

All neural network code was based on PyTorch [36]. Training was performed in a similar manner for all three test cases. We used the Adam optimizer with a cosine warm-up learning rate scheduler with 50 warm-up steps, as described in [37]. That is, the learning rate started as a small value, and was increased to the chosen value over 50 epochs. Thereafter, it was reduced following a cosine function over the remaining epochs. The gradient norms were clipped to 0.5, to stabilize the training. We made use of early stopping with a patience of 50 to avoid over-fitting; that is, if there were 50 consecutive epochs without improvement on the validation data, we stopped the training and made use of the best performing model. In all test cases, we trained on 25,000 samples and used 5000 samples for validation. For all hyperparameters, see Table A1.

**Table A1:** Hyperparameters for the WAEs.

	WDN 1	WDN 2	WDN 3
**Training hyperparameters**			
Batch size	256	256	256
Learning rate	$5\times 10^{-5}$	$5\times 10^{-5}$	$5\times 10^{-5}$
$l^{2}$ regularization	$10^{-10}$	$10^{-10}$	$10^{-10}$
MMD regularization	$10^{-2}$	$10^{-2}$	$10^{-2}$
Scheduler warmup	50	50	50
Early stopping patience	50	50	50
**ResNet Architecture**			
Latent dimension	8	16	16
# layers	5	5	7
# neurons	64, 48, 32,	192, 160, 128,	512, 384, 320,
	24, 16	96, 64, 32	256, 192, 128, 64
Act. func.	Leaky ReLU	Leaky ReLU	Leaky ReLU
**Transformer Architecture**			
Latent dimension	8	16	16
Num. dense neurons	32	128	256
Embedding dimension	4	4	4
# Attn. heads	2	2	2
# Latent transformer blocks	2	2	2
# Full order transformer blocks	1	1	1
Act. func. transformer layers	GeLU	GeLU	GeLU
Act. func. dense layers	Leaky ReLU	Leaky ReLU	Leaky ReLU

## Appendix D. Classification Model

The classification neural network, *g*, outputs a vector of the same size as the number of pipe sections, $N_{p}$. Furthermore, it has a softmax activation after the last layer, to ensure that the output sums to 1. The model was trained on a labeled dataset consisting of noiseless sensor observations, H(x)=y and the corresponding leak location, *c*, as the target. The leak location was one-hot encoded; that is, the target was a zero vector with a one at the entry of leak location index. Hence, the dataset is given by

(A2) $$\begin{array}{c} \left\{\left(H\left(x_{1}\right),c_{1}\right),\ldots ,\left(H\left(x_{N}\right),c_{N}\right)\right\}. \end{array}$$

The neural network is trained using the cross-entropy loss:

(A3) $$\begin{array}{c} L\left(g\right)=-\sum_{i=1}^{N}\sum_{j=1}^{N_{p}}c_{i,j}\log(g\left(H{\left(x_{i}\right)}_{j}\right), \end{array}$$

where $c_{i,j}$ is the *j*th index of the *i*th training sample and $H{\left(x_{i}\right)}_{j}$ is the *j*th index of the output of the neural network evaluated at the *i*th training sample. Hence, the neural network was trained to output the probability of a leak being present in each possible pipe section given the observations, i.e., the posterior distribution, $g\left(y_{i}\right)=p_{c|y}\left(c_{k}|y_{i}\right)$. In the online stage, the distribution is updated with observations in time, by

(A4) $$\begin{array}{c} p_{c|y}\left(c_{k}|y_{0:N_{t}}\right)=p_{c}\left(c_{k}\right)\prod_{i=0}^{N_{t}}p_{c|y}\left(c_{k}|y_{0:i}\right)=p_{c}\left(c_{k}\right)\prod_{i=0}^{N_{t}}g\left(y_{i}\right), \end{array}$$

until convergence with respect to the KL-divergence. This is the same procedure as for the proposed framework.

It is important to keep in mind that the classifier only approximates the leak location posterior for a single sensor configuration. This is in contrast to the proposed framework, where the full state posterior is approximated together with the leak location. This also means that the classification neural network needs to be retrained every time the sensor configuration is changed.
